# Action Monitoring Cortical Activity Coupled to Submovements

**DOI:** 10.1523/ENEURO.0241-17.2017

**Published:** 2017-10-24

**Authors:** Michael Pereira, Aleksander Sobolewski, José del R. Millán

**Affiliations:** Chair in Brain-Machine Interface, Center for Neuroprosthetics, Swiss Federal Institute of Lausanne, Geneva CH-1202, Switzerland

**Keywords:** EEG, error, kinematics, monitoring, submovements, supplementary motor area

## Abstract

Numerous studies have examined neural correlates of the human brain’s action-monitoring system during experimentally segmented tasks. However, it remains unknown how such a system operates during continuous motor output when no experimental time marker is available (such as button presses or stimulus onset). We set out to investigate the electrophysiological correlates of action monitoring when hand position has to be repeatedly monitored and corrected. For this, we recorded high-density electroencephalography (EEG) during a visuomotor tracking task during which participants had to follow a target with the mouse cursor along a visible trajectory. By decomposing hand kinematics into naturally occurring periodic submovements, we found an event-related potential (ERP) time-locked to these submovements and localized in a sensorimotor cortical network comprising the supplementary motor area (SMA) and the precentral gyrus. Critically, the amplitude of the ERP correlated with the deviation of the cursor, 110 ms before the submovement. Control analyses showed that this correlation was truly due to the cursor deviation and not to differences in submovement kinematics or to the visual content of the task. The ERP closely resembled those found in response to mismatch events in typical cognitive neuroscience experiments. Our results demonstrate the existence of a cortical process in the SMA, evaluating hand position in synchrony with submovements. These findings suggest a functional role of submovements in a sensorimotor loop of periodic monitoring and correction and generalize previous results from the field of action monitoring to cases where action has to be repeatedly monitored.

## Visual Abstract

**Figure:**
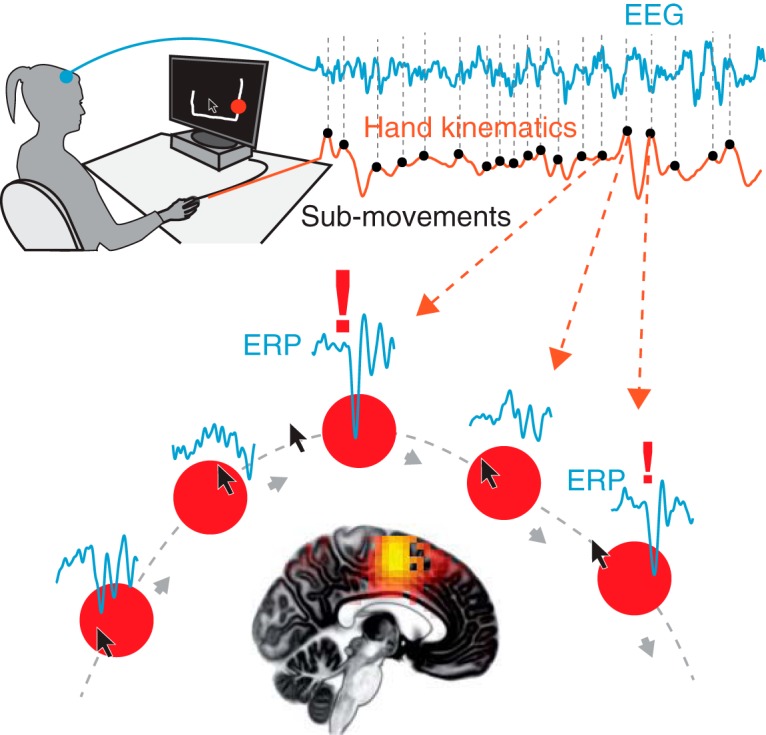

## Significance Statement

Monitoring the effect of our actions to correct them is a key function of the brain for adaptive behavior. We investigated how such an action-monitoring system operates in continuous, visually-guided movements, when hand position has to be repeatedly monitored and corrected. We show that during such movements, an electrophysiological process occurs in synchrony with periodically occurring pulses in hand kinematics (submovements). Crucially, the amplitude of the corresponding electrophysiological markers was correlated with the deviation of the hand. Our findings show that during continuous movements, the action-monitoring system of the brain is synchronized with periodic submovements. Moreover, we provide neural evidence supporting a functional role of (low-frequency) cortical activity synchronized to motor output

## Introduction

Our brain needs to constantly monitor the consequences of the actions it generates to correct for erroneous actions. Neural correlates of such an action-monitoring system have been repeatedly found in the medial frontal cortex (Ridderinkhof et al., 2004; Debener et al., 2005). Electrophysiological studies have found error-related activity after erroneous button presses (Falkenstein et al., 1991; Gehring et al., 1993) and, more recently, after perturbations during rapid goal-directed movements (Vocat et al., 2011; Torrecillos et al., 2014). Abundant work on the neural correlates of errors thus uncovered much of the functioning of the brain’s action-monitoring system. However, they have been mostly constrained to single, well-defined events such as button presses or fast reaching movements. Conversely, much of human behavior and resulting visual feedback is a seemingly continuous and not easily parsed operation (Cisek and Kalaska, 2010). Here, we study electrophysiological correlates of action monitoring in continuous, visually-guided movements.

During such movements, the kinematics and electromyographical (EMG) activity of the upper limb reveal a succession of bell-shaped pulses or “submovements,” with periodicities between 2 and 10 Hz, depending on the muscles involved (Vallbo and Wessberg, 1993; Jerbi et al., 2007; Williams et al., 2010). Behavioral studies have shown that the magnitude of these submovements corresponds to deviations from the desired position, indicating their error-related or corrective nature (Miall et al., 1986; Selen et al., 2006). However, although some studies have found electrophysiological correlates of perturbations during reaching movements (Archambault et al., 2009; Torrecillos et al., 2014; Dipietro et al., 2015), to the best of our knowledge, no brain correlate of error processing has been linked to periodic endogenous submovements so far. Moreover, none of the above studies dissociated error processing from differences in kinematics.

Our aim was to show how the brain’s action-monitoring system operates during continuous movements, when actions have to be repeatedly monitored and no experimental time marker is available. For this, 23 healthy participants used a mouse cursor to follow a moving target on the computer screen (tracking condition). The trajectory followed by the target was visible and was drawn by the subjects themselves in a previous condition (spontaneous condition). Additionally, after half of the tracking trials, a replay of the trial was shown to the subjects as a control condition (viewing condition): subjects watched (without moving) the target and the mouse cursor moving on the screen. We recorded high-density electroencephalography (EEG) and thereby report an event-related potential (ERP) time-locked to the submovements. We then compared the amplitude of these ERPs with the deviation of the cursor relative to the target, while controlling for motor confounds. We found that the ERP was modulated by cursor deviation, 110 ms before the submovement, irrespectively of hand kinematics.

## Materials and Methods

### Subjects

Twenty-three right-handed healthy subjects (seven women) participated in the study. Subjects were aged between 20 and 30 years, with normal or corrected-to-normal vision. They had no reported neurologic or psychiatric problems. The study was approved by the local university ethics committee and all participants gave written informed consent.

### Experimental protocol

Subjects performed 20 times the following sequence of tasks. First, participants were instructed to move the computer mouse at a constant speed for 20 s to create a spontaneous curvilinear trajectory (“spontaneous” condition). This trajectory was spatially restricted to an area of the (24’’) computer screen subtending a 20° horizontal and 13° vertical visual angle, corresponding to 840 by 525 pixels (px). The unfolding trajectory was not drawn on the screen: only a cursor was visible to the subjects. Subjects were compelled to keep a steady pace by having to repeat trials exceeding speed limits. Their pace was automatically monitored by our software. Additionally, apparent speed of the mouse cursor was kept under 250 px/s by a smoothing algorithm, applied in real-time during the spontaneous condition.

Spontaneous trials were followed by a visuomotor tracking task (“tracking” condition): the previously generated trajectory was shown on the screen and a target (a red circle of 15-px radius) moved along it replicating the movement recorded during the preceding spontaneous trial (after the real-time smoothing). The rationale behind showing the trajectory was to study motor errors rather than surprising changes in target position. The participants were instructed to track the target with a standard computer mouse driving a typical cursor (an arrow), keeping it as close to the target’s center as possible. At the end of each trial, a score ranging from 0 to 100 was displayed as an incentive to perform well. The score was based on a linear transformation of the mean distance between the cursor and the target center. Subjects used their right hand to operate the computer mouse in both conditions. Finally, after half of the tracking trials, a replay of the preceding trial was shown to the subjects as a control condition (“viewing” condition): subjects watched (without moving) the target and the mouse cursor move on the screen as recorded during the preceding tracking condition. This additional control was used in only half of the trials to reduce the duration of the experiment.

### Behavioral measures

As opposed to discrete action monitoring paradigms, our tracking experiment allowed for continuous behavioral variables to be measured. We recorded mouse cursor and target positions at 50 Hz (the refresh rate of the monitor) and interpolated these data offline (using piecewise cubic interpolation) to match the 256 Hz sampling rate of the preprocessed EEG. Two measures were derived from these positional data.

**Figure 1.**
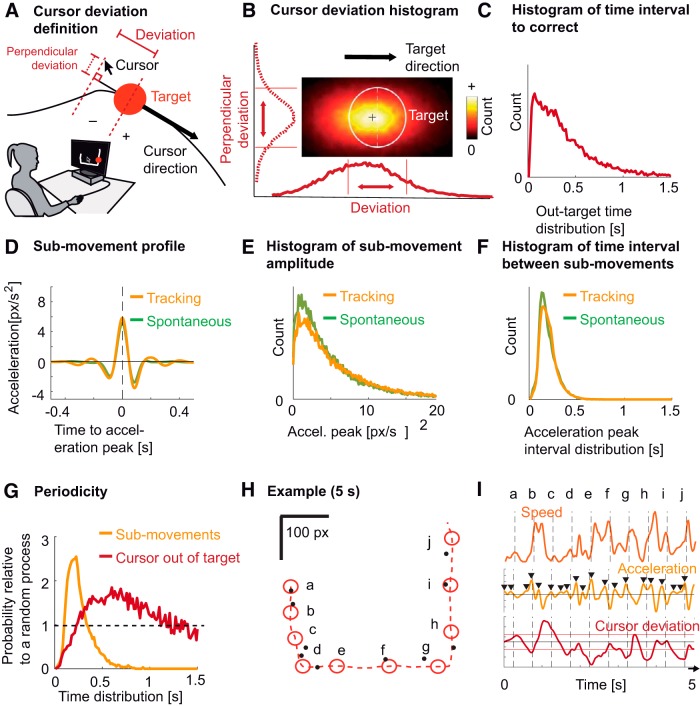
Cursor deviation and hand kinematics (submovements). ***A***, Experimental setup and definition of cursor deviation. Subjects used a mouse cursor (a typical arrow) to track a target (red circle) moving on a computer screen along a visible trajectory. Cursor deviation was defined as the cursor’s position relative to the position of the target along the tangent to the target’s direction. Perpendicular deviation was defined as the deviation relative to the tangent. ***B***, Distribution of cursor deviations around the target (white circle), averaged across subjects. The lower curve corresponds to the distribution of the cursor deviation used in the rest of the manuscript. The left (dashed) curve corresponds to the perpendicular deviation. Red vertical lines indicate target borders. ***C***, Distribution of the time needed to correct cursor deviations: from the time the cursor left the target area to the time it went back in, averaged across subjects. ***D***, Average hand acceleration profile, time-locked to submovements for tracking (yellow trace) and for spontaneous tracing (green trace), averaged across subjects. ***E***, Distribution of the magnitude of hand acceleration peaks for tracking (yellow trace) and spontaneous tracing (green trace), averaged across subjects. ***F***, Distribution of time intervals between submovements for tracking (yellow trace) and spontaneous tracing (green trace), averaged across subjects. ***G***, Probability of time intervals relative to a random (Poisson) process for two types of events: submovements (yellow trace) and cursor leaving the target area (red trace). ***H***, An example of 5 s of tracking. The target is depicted by red circles shown every 500 ms (a-j). The dashed red trace shows the trajectory of the target, moving from a to j. The corresponding cursor positions are depicted by black dots. ***I***, Hand speed (orange trace), hand acceleration (yellow trace), and cursor deviation (dark red trace) for the 5 s depicted in ***H***. Peaks selected by the peak selecting algorithm are depicted by black triangles. Horizontal red lines show target borders. Vertical dashed lines correspond to target positions in ***H***.

Firstly, we quantified the subjects’ instantaneous performance using the distance between the target center and the mouse cursor, projected onto a line tangential to the trajectory of the target (Fig. 1*A*). This cursor deviation measure was then smoothed using a quadratic Savitzky-Golay filter with a 0.106-s window (Savitzky and Golay, 1964). The absolute value of this cursor deviation measure explained most of the variance of the more intuitive Euclidian distance between cursor and target center (*R* ^2^ = 0.84 ± 0.01 on average). However, we assumed that the brain uses a more functional deviation measure that can be directly translated into the amount of correction needed.

Secondly, to decompose the subjects’ hand kinematics into submovements to align our ERP analysis, we computed the acceleration of the hand (or cursor). We first computed hand velocity by differentiating consecutive hand positions with a quadratic Savitzky-Golay filter and rectifying to obtain the hand speed profile. We then differentiated the speed profiles using a quadratic Savitzky-Golay derivative filter to obtain the hand acceleration profile. We set the window length of the smoothing filter to 0.106 s as we found this was an optimal balance between efficiently removing high frequency spurious peaks while keeping the spectral structure. Submovements were defined as peaks in the hand acceleration profiles, i.e. samples higher than their neighbors and higher than zero. Since hand acceleration is closely related to EMG (Vallbo and Wessberg, 1993), we assumed that these peaks were the best available markers of submovements.

### Electrophysiological recording and processing

Scalp EEG activity was recorded from 64 active electrodes in an extended 10–20 layout using a Biosemi ActiveTwo system and digitized at 2048 Hz. Data were down-sampled off-line to 256 Hz, rereferenced to a common average reference and bandpass filtered (Butterworth; zero-phase two-pass) between 1 and 15 Hz (3 dB cutoff). To verify that the filtering did not induce any distortion, we replicated the findings without filtering but using only de-trending of each one second epoch and obtained similar results. Electroocular artifact were removed (see Artifact rejection section). EEG data were then segmented into one-second epochs, each centered around one acceleration peak. ERP were obtained by averaging epochs and averaging the resulting waveform across subjects. Mean amplitudes were computed by taking the mean of the ERP in a 0.1 s time interval centered around the latency of the ERP trough. This method is considered to be robust against noise (Clayson et al., 2013) and was used for both single-trial measurements and ERP amplitude measurement of individual subjects.

### Artifact rejection

Although smooth pursuit of the target is the natural ocular behavior during visuomotor tracking at low speed (Miall et al., 1993), we took great care in excluding any possible effect of eye movement artefacts on the results. Firstly, the instructions to the subjects to keep the speed of the mouse cursor low during the spontaneous condition and the real-time speed smoothing helped prevent possible saccadic eye movements. Secondly, EOG data were recorded with three sensors, placed above the nasion and below the outer canthi of the participants’ eyes. Horizontal EOG (hEOG) was defined as the difference between signal from the outer canthi sensors and vertical EOG (vEOG) activity as the difference between the nasion and the mean of the outer canthi signals. All parts of the signal containing EEG, vEOG or hEOG amplitudes larger than 50 µV were discarded from further analyses. Furthermore, to ensure that no small EOG component (such as saccades) could influence our results, we regressed out hEOG and vEOG signals (bandpass filtered with the same filter as for the EEG) from the remaining EEG signals.

### Single-epoch amplitude map

To explore the ERP’s relationship to task performance, we computed the amplitude of the EEG single-epochs (FCz electrode) and binned these amplitudes according to the cursor deviation. Since this cursor deviation could be measured at various latencies with respect to the acceleration peak, we could not know a priori at which latency the brain samples end-effector deviation. Therefore, for every sampling times (0.01 s bins, range: [−0.3, 0.3] s around the acceleration peak; dimension 1), we binned single-trial ERP amplitudes according to cursor deviations from −60 to +60 px into 1 px wide bins (dimension 2). The resulting two-dimensional deviation-latency map of single-epoch amplitudes was smoothed along the spatial dimension using a Gaussian kernel (2.5 px SD). However, not all the subjects had the same cursor deviation distribution so we restricted the displayed area of the so-obtained map in a way that all points in the map corresponded to a minimum of 40 single-trial measurements for every subject.

If no relationship existed between single-trial amplitudes and the cursor deviation, we expected the map to be flat, not showing any systematic pattern. On the contrary, if the ERP was modulated by cursor deviation occurring before the submovement onset (acceleration peak), we expected to see larger amplitude differences in the left part of the map. If the ERP was modulated by cursor deviation occurring after the submovement, the map should reflect this with larger amplitude differences in the right part.

### Matching kinematics

To obtain sets of epochs with similar kinematics between either experimental conditions or cursor deviation bins, we constructed a matrix of pairwise mean-square errors (MSEs) between epochs of each condition and iteratively selected (without replacement) pairs with the smallest MSE until a threshold number of paired epochs was achieved (or all epochs from one condition were included).

### Phase-locking to behavioral events

To confirm that our ERP was coupled to submovements and not to any visual event, we assessed phase-locking for four different types of events. Phase-locking is preferable over comparing ERP amplitudes since it dissociates phase from amplitude contributions. Since the ERP has a low-frequency support (around 5 Hz), any event underlying the ERP should be associated with a significant phase modulation at this frequency range. The phase was computed by bandpass filtering (Butterworth two-pass zero-phase) between 3 and 7 Hz (3 dB cutoff), correcting for EOG (see Artifact rejection section) and applying a Hilbert transform. Phase-locked values (PLVs) were extracted at each behavioral event. To control for different number of events and non-genuine phase-locking, the PLV was normalized (*z* score) using the mean and SD of 1000 surrogate PLV computed by randomly shifting the behavioral data with respect to the EEG. It was then possible to assess whether these z-scored PLV (zPLV) were consistently different from zero across subjects with a one-sample *t* tests.

### Statistics

Because of the exploratory nature of the study, the α level was set to 0.01, except for the control experiment that was hypothesis driven for which the α level was set to 0.05. Bonferroni corrections were applied when necessary. Post-hoc achieved power was computed with the G*Power software (Faul et al., 2007) and reported in Table 1. In the case of multiple comparisons, the power of the test returning the minimum p-value is reported with the α level adjusted (divided by the number of multiple comparisons). For the two-way repeated measures ANOVA, we estimated the power of each of the two main effects independently, using two one-way repeated measures ANOVA.

**Table 1.** Statistical table

	Data structure	Type of test	Power
a	ERP data for each subject (*N* = 23), repeated for each time point (*N* = 257)	One-sample *t* test for all time samples (*N* = 257), Bonferroni corrected	1
b	ERP data for each subject (*N* = 23), repeated for each time point (*N* = 257)	One-sample *t* test for all time samples (*N* = 257), Bonferroni corrected	1
c	ERP data for each subject (*N* = 23), repeated for each time point (*N* = 257)	One-sample *t* test for all time samples (*N* = 257), Bonferroni corrected	0.73
d	Mean amplitude of ERPs for each subject (*N* = 23) for two tasks (track/spont.)	Repeated measures ANOVA with factor task	0.7
e	Mean amplitude of ERPs for each subject (*N* = 23) for seven levels of cursor deviation	Repeated measures ANOVA with factor deviation	0.16
f	Mean amplitude of ERPs across subjects for 7 levels of cursor deviation	*F* test	1
g	Mean amplitude of ERPs across subjects for 7 levels of cursor deviation	*F* test	0.41
h	Mean amplitude of ERPs for each subject (*N* = 23) for tracking and spontaneous (repeated for 7 levels of deviation)	Paired *t* test for all cursor deviation bins (*N* = 7), Bonferroni corrected	0.96
i	Mean amplitude of ERPs across subjects for 7 levels of cursor deviation	*F* test	1
j	Mean zPLV for each subject (*N* = 23)	One-sample *t* test	0.07
k	Mean zPLV for each subject (*N* = 23)	One-sample *t* test	0.07
l	Mean zPLV for each subject (*N* = 23)	One-sample *t* test	0.95
m	Mean zPLV for each subject (*N* = 23)	One-sample *t* test	1
n	Mean zPLV for each subject (*N* = 23)	Paired *t* test	1
o	Mean amplitude of ERPs across subjects for 7 levels of cursor deviation	*F* test	1*

*p = 0.05 instead of *p* = 0.01.

## Results

### Subjects failed to keep the mouse cursor inside the target

We quantified instantaneous task performance as the cursor’s position projected onto the tangent to the target’s direction (Fig. 1*A*). Our measure allowed discriminating between deviations consisting in the cursor falling behind the target (negative values) and overtaking it (positive values). The cursor was behind the target 35 ± 6% (mean ± SEM across subjects) of the time and ran ahead 25 ± 5% of the time; the remaining 40 ± 5% of the time, the cursor was inside the target. Fig. 1*B* shows the distribution of cursor deviations parallel to the target direction (solid line) and perpendicular to the target direction (dashed line). The mean distance between the cursor and the trajectory (7.4 ± 0.18 px) was much lower than the average Euclidian distance to the target center (20.6 ± 0.5 px), suggesting that subjects were good at following the trajectory but failed to keep up with the target position along the trajectory. On average, subjects spent 0.322 ± 0.010 s outside the target’s area before successfully correcting the deviation (Fig. 1*C*).

### Hand kinematics are composed of periodic submovements

To represent submovements, we used hand acceleration, computed from hand positions recorded during the task. Consistent with earlier studies (Vallbo and Wessberg, 1993), these kinematics were composed of successive submovements, which were not due to the curvature of the trajectories (Fig. 1*H*,*I*). Submovements were defined as peaks in the hand acceleration to which we aligned all subsequent analyses. The resulting averaged profile of submovement showed a triphasic wave form which was similar between the tracking and the spontaneous condition (Fig. 1*D*). For both conditions, the peak of the acceleration showed an exponentially decreasing distribution (Fig. 1*E*). The median time interval between two submovements was 0.200 ± 0.002 s for tracking and 0.195 ± 0.002 for spontaneous tracing, corresponding to a frequency of 5.0 and 5.1 Hz, respectively (Fig. 1*F*).

To verify the periodicity of submovements, we normalized the distribution of the time intervals between two consecutive submovements by the theoretical distribution expected from a random (Poisson) process with identical rate. This measure thus quantifies how much more probable is a time interval between two consecutive submovements compared to a random process. A consistent peak was found for the tracking condition (2.55 times more probable than a random process; maximum at 0.200 ± 0.007 s; Fig. 1*G*). On the other hand, the same analysis applied to the times when the cursor leaves the target area showed a lower and more smeared peak (1.84 times more probable than a random process; maximum at 0.72 ± 0.035 s). Finally, Fig. 1*H* shows an example of 5 s of tracking with cursor and target positions marked every 0.5 s along with the corresponding deviation and kinematics metrics (Fig. 1*I*).

### Existence of an ERP locked to submovements

**Figure 2.**
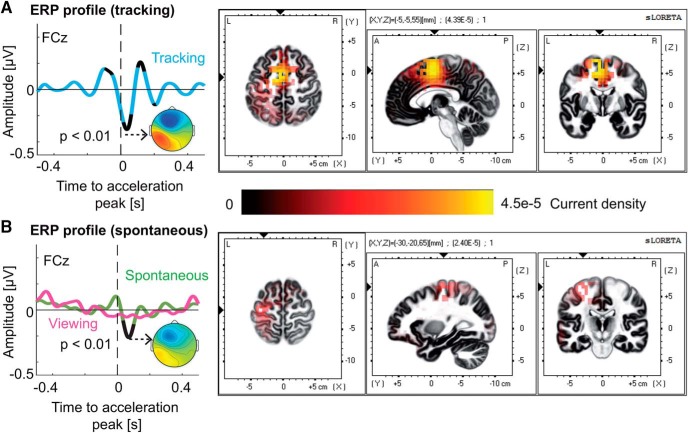
ERP time-locked to submovements ***A***, The ERP time-locked to submovements for the tracking (cyan trace) conditions, averaged across subjects. The ERP showed a significant trough localized in the medial frontal gyrus (right inset). Significant portions of the ERP are shown in black (*p* < 0.01, Bonferroni corrected). ***B***, The ERP time-locked to hand acceleration for the spontaneous (green trace) and viewing (magenta trace) conditions, averaged across subjects. During the spontaneous condition, the ERP showed a significant trough (black segment, *p* < 0.01, Bonferroni corrected), localized in the left (contralateral) precentral gyrus. The ERP for different directions of the target (orange for north, blue for east, purple for south and yellow for west; see inset) can be found in Extended Data Figure 2-1. No discernible differences in ERP amplitude were observed.

We then investigated the existence of electrophysiological activity locked to submovements. By averaging one second epochs of EEG centered around submovements, we found a significant ERP (FCz; *p* < 0.01^a^, *t*_(22)_ < −5.19; Fig. 2*A*, left). The ERP mainly consisted of a negative peak (trough), 0.038 ± 0.003 s after submovement onset. For the spontaneous condition, a smaller yet significant (FCz; *p* < 0.01^b^, *t*_(22)_ < −5.20; Fig. 2*B*, left) negative ERP wave was found, reaching its trough 0.070 ± 0.003 s after the acceleration peak.

We then used exact low-resolution brain tomography (eLORETA) to locate the sources of the ERP (Pascual-marqui, 2007). For tracking, the trough of the ERP had its strongest source in the medial frontal gyrus, Brodmann area 6 Montreal Neurological Institute (MNI) coordinates: X = −5, Y = −5, Z = 55, corresponding to the left supplementary motor area (SMA) using the automatic anatomic labeling atlas; Tzourio-Mazoyer et al., 2002; Fig. 2*A*, right]. In the spontaneous condition, the sources of the ERP were strongest in the left precentral gyrus, Brodmann area 4 (MNI: X = −30, Y = −20, Z = 65; Fig. 2*B*, right). This region also showed activation in the tracking condition.

To control for the influence of pure visual input on the ERPs, we also repeated the ERP analysis using data from the viewing condition during which subjects were simply watching their performance recorded in the preceding tracking task (identical visual stimulation). The analysis was thus aligned to peaks in cursor acceleration. No ERP was found for the visual condition (FCz; *p* > 0.087^c^, *t*_(22)_ > −2.49; Fig. 2*B*, left). These results, in addition to our EOG correction and the absence of discernable differences due to target-direction (Extended Data Fig. 2-1) allow us to assert that neither the pure visual input without the behavioral context, nor EOG artefacts, were the origin of the electrophysiological phenomenon described herein.

10.1523/ENEURO.0241-17.2017.f2-1 Supplementary Figure 2-1 The ERP for different directions of the target (orange for north, blue for east, purple for south and yellow for west; see insert) can be found in Figure 2-1. No discernible differences in ERP amplitude were observed. Download Figure 2-1, EPS file.

### Latency of the influence of cursor deviation

**Figure 3.**
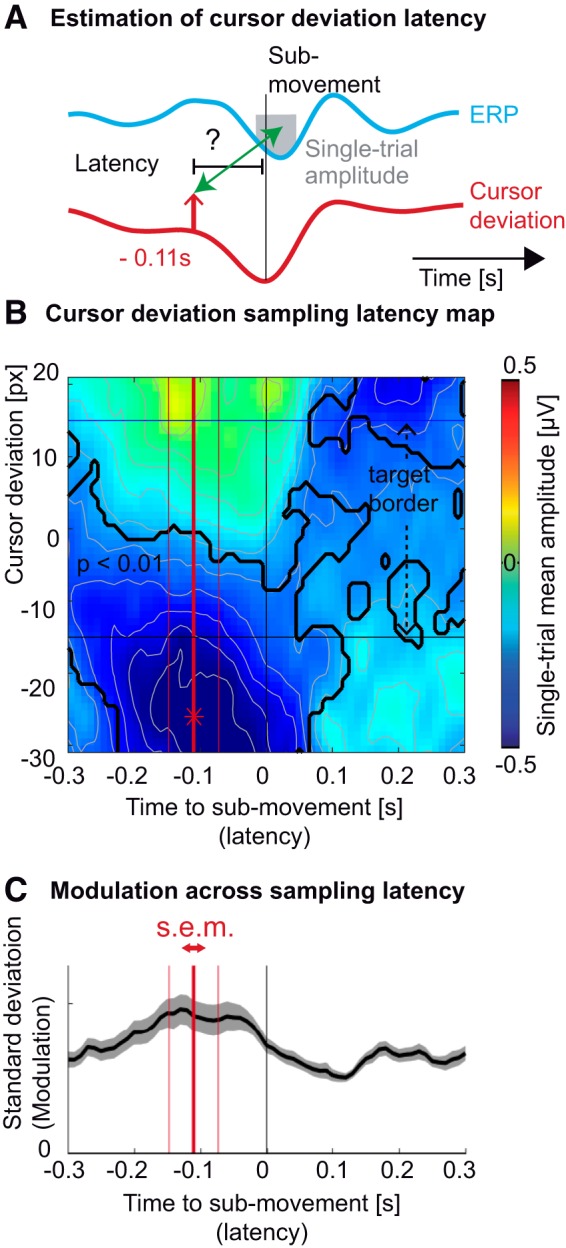
Latency of the sampling of cursor deviation. ***A***, Average profile of the ERP (cyan trace) and cursor deviation (red trace), time-locked to hand acceleration peaks. The upward pointing arrow depicts one potential sampling latency of the cursor deviation, possibly influencing the amplitude of ERP (gray box). ***B***, Averaged single-epoch mean amplitude (FCz electrode) as a function of the cursor deviation (vertical axis) and the sampling latency of the cursor deviation relative to the submovement (horizontal axis). The largest trough (depicted by a red asterisk) was observed for the cursor markedly lagging behind the target (negative cursor deviation) before the acceleration peak. The black trace shows portions of the map corresponding to ERP amplitudes significantly different from zero across subjects (*p* < 0.01, Bonferroni corrected). The horizontal black lines represent the target borders. ***C***, The SD of the ERP amplitudes across cursor deviation. The maximum value, averaged across subjects (thick red vertical bar) of the minimum single-trial amplitude, was estimated to be 0.11 s before the submovement. The two thin red vertical bars represent the SEM of this estimation.

Following the goal of our study, we sought to investigate the relationship of the ERP to behavioral performance, i.e. cursor deviation. However, we did not know a priori the latency with respect to the submovement at which the cursor deviation would, hypothetically, modulate the amplitude of the ERP (Fig. 3*A*). We thus constructed a two-dimensional map of single-trial amplitudes depending on the cursor deviation and the latency at which it occurred with respect to the submovement.

The resulting map showed that the largest troughs (negative amplitude, i.e. large ERP trough; blue color) were observed for cursor deviations behind the target, occurring 0.11 s before the submovement (Fig. 3*B*). This latency was further confirmed by analyzing which latency around the submovements led to the largest SD of the ERP amplitudes across cursor deviations.The largest ERP modulation occurred at a latency of 0.11 ± 0.04 s before the submovements’ acceleration peaks (Fig. 3*C*). Informed by the results of this exploratory analysis, we sought to verify them, controlling for the possible influence of varying hand kinematics.

### Modulation of the ERP by cursor deviation

**Figure 4.**
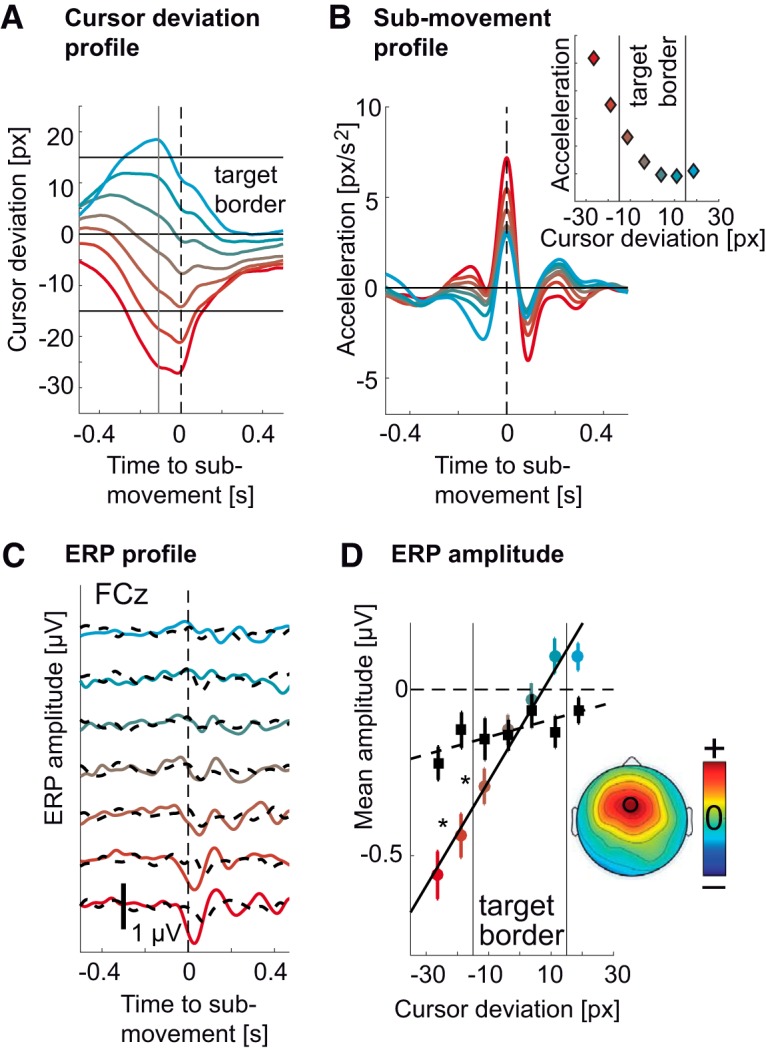
Modulation of the ERP by cursor deviation. ***A***, Colored traces correspond to different average cursor deviation time courses leading to ERPs in ***C*** for the tracking condition. Red traces correspond to deviations occurring behind the target and cyan traces correspond to deviations occurring ahead of the target. The same coding scheme was used throughout the figure. The vertical gray line corresponds to the sampling of the error at 0.11 s before the submovement, leading to the modulation of the ERP. Horizontal black lines indicate the target’s center and borders. ***B***, Average hand submovement kinematics (acceleration) corresponding to cursor deviations in ***A*** and ERPs in ***C*** for the tracking condition. Increasing lagging of the cursor behind the target led to increasing submovement kinematics (to catch up with the target). The panel on the upper right shows the relation between cursor deviation and submovement peak acceleration. The results of the matching procedure can be found in Extended Data Figure 4-1 with colored traces showing kinematic profiles of submovements from the tracking condition and dashed black traces corresponding to matched submovements from the spontaneous condition. Panels are ordered from left to right in order of increasing cursor deviation. ***C***, ERP (FCz) for different cursor deviations (and submovement kinematics) showed increasing amplitudes for increasing lag of the cursor behind the target (negative values). ***D***, Amplitude of the ERP troughs (FCz) from ***C*** against cursor deviation at 0.11 s presubmovement. The colored dots correspond to ERP amplitudes from the tracking condition and were correlated with cursor deviation (*r*^2^ = 0.97, *p* < 0.001). The black squares correspond to ERP amplitudes from the spontaneous condition and also linearly increased (*r*^2^ = 0.63, *p* = 0.034). The amplitudes were computed by averaging the ERP in time over a 0.1-s window centered on the ERP trough. More negative values correspond to larger troughs. Whiskers denote SEM across subjects. The asterisks show significant differences between the tracking and the spontaneous condition (*p* < 0.05, Bonferroni corrected). Vertical black lines indicate the target’s border. For the tracking condition, the slope of this linear fit was strongest over the frontal midline (inset).

To verify that hand kinematics are not the main factor of ERP modulation, we divided the EEG epochs of the tracking condition into bins according to the cursor deviation 0.11 s before the acceleration peak. For each bin, we selected a subset (*N* = 100) of epochs for which we could find an equal number of epochs in the spontaneous condition that showed maximal similarity in terms of hand kinematics (Extended Data Fig. 4-1). For the tracking condition, different bins showed different cursor deviation (Fig. 4*A*), corresponding to different submovement kinematics (Fig. 4*B*). These selected submovements showed increasing acceleration as the cursor lagged behind the target but similarly low acceleration when the cursor was in front of the target center (Fig. 4*B*, inset).

10.1523/ENEURO.0241-17.2017.f4-1 Supplementary Figure 4-1 The results of the matching procedure can be found in Figure 4-1 with colored traces showing kinematic profiles of sub-movements from the tracking condition and dashed black traces corresponding to matched sub-movements from the spontaneous condition. Panels are ordered from left to right in order of increasing cursor deviation. Download Figure 4-1, EPS file.

For the tracking condition, the ERP was larger for cursor deviations behind the target. The ERP in the spontaneous condition, which did not correspond to any cursor deviation but had identical kinematics showed a much reduced modulation (Fig. 4*C*). We quantified these ERP amplitudes by computing the mean of the ERP for every subject in a 0.1-s window centered around the ERP trough’s latency (Clayson et al., 2013). A repeated measures ANOVA on the so-computed ERP amplitudes revealed a significant effect of cursor deviation (*F*_(6,132)_ = 16.42, *p* < 0.001^d^), but not of task (tracking versus spontaneous; *F*_(1,22)_ = 2.79, *p* = 0.11^e^). However, there was a significant interaction between task and cursor deviation (*F*_(6,132)_ = 11.86, *p* < 0.001). Post-hoc tests showed that the amplitude of the ERP was significantly correlated to the cursor deviation (*r* ^2^ = 0.97, *p* < 0.001^f^, *F*_(1,6)_ = 141.05; Fig. 4*D*), strongest above the frontal midline (Fig. 4*D*, inset). For the spontaneous condition, the correlation was much weaker (*r* ^2^ = 0.63, *p* = 0.034^g^, *F*_(1,6)_ = 8.32). There were also significant differences in ERP amplitudes between the tracking and the spontaneous condition (*p* < 0.01^h^, Bonferroni corrected).

### Modulation by cursor deviation is independent from hand kinematics

**Figure 5.**
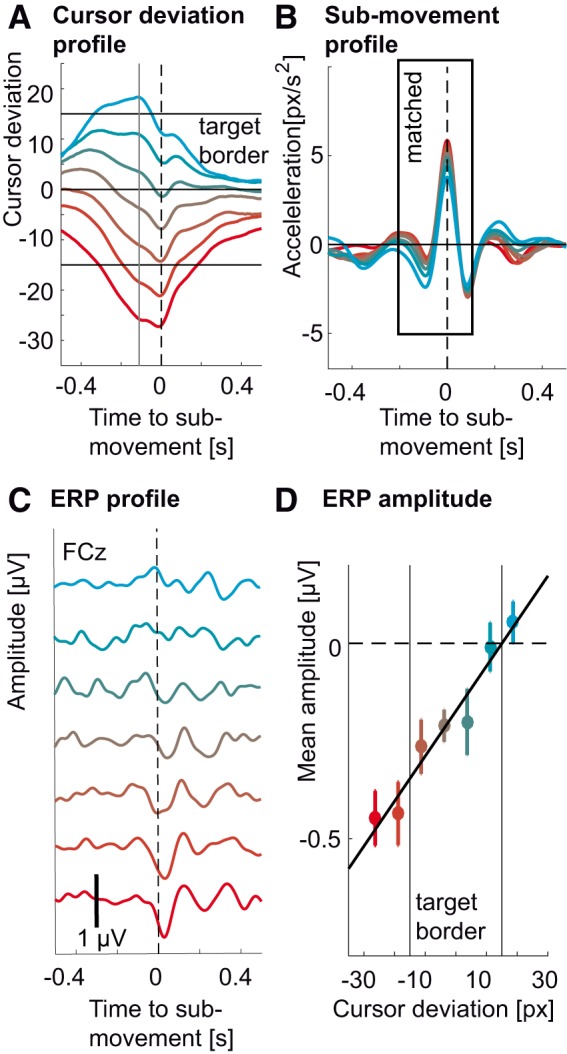
Modulation of the ERP by cursor deviation in the tracking task while controlling for kinematics. ***A***, Colored traces represent average cursor deviation time courses leading to ERPs in ***C***. Red traces correspond to deviations occurring behind the target and cyan traces correspond to deviations occurring ahead of the target. The same coding scheme was used throughout the figure. The vertical gray line corresponds to the sampling of the error at 0.11 s before the submovement leading to the modulation of the ERP. Horizontal black lines indicate the target’s center and borders. ***B***, Matched average submovement kinematics (acceleration) corresponding to cursor deviations in ***A*** and ERPs in ***C***. ***C***, Average ERPs (FCz) for different cursor deviations during the tracking task (colored traces) for matched hand kinematics. The size of the ERP increased with increasing lag of the cursor behind the target (negative values). ***D***, Amplitude of the ERP troughs (FCz) from ***C*** correlating to cursor deviation at 0.11 s presubmovement (*r* ^2^ = 0.95, *p* < 0.001). The amplitudes were computed by averaging the ERP in time over a 0.1-s window centered on the ERP’s trough. Negative values correspond to larger troughs. Whiskers denote SEM across subjects. Vertical black lines indicate the target’s border. The peak-to-peak ERP amplitudes for the control experiment with constant target speed are in Extended Data Figure 5-1*A*. Vertical black lines indicate target borders. Kinematics (top) and cursor deviation profiles corresponding to the ERP amplitudes are in Extended Data Figure 5-1*B*.

Since binning the cursor deviation led to differences in hand kinematics, we sought to repeat the analysis controlling for this confound (low frequencies in EEG are known to carry correlates of motor behavior; Waldert et al., 2008). Therefore, we divided the EEG epochs into the same bins according to the cursor position 0.11 s before the acceleration peak. However, we used only a subset of epochs (*N* = 80) that showed the most similar acceleration profiles across bins. Using this method, we were able to keep hand acceleration profiles similar (Fig. 5*B*) across different deviations (Fig. 5*A*). The ERP were still larger for cursor deviations behind the target (Fig. 5*C*), with amplitudes significantly correlated to cursor deviation (*r* ^2^ = 0.95, *p* < 0.001^i^, *F*_(1,6)_ = 97.54; Fig. 5*D*).

### Control analyses

Since our task comprised many inter-dependent variables such as differences in target speed (target acceleration), cursor deviation and hand kinematics, we performed two control analyses to verify that the ERP was (1) truly coupled to hand-kinematics and not to target acceleration or cursor deviation and (2) truly related to cursor deviation and not to unexpected differences in target speed.

**Figure 6.**
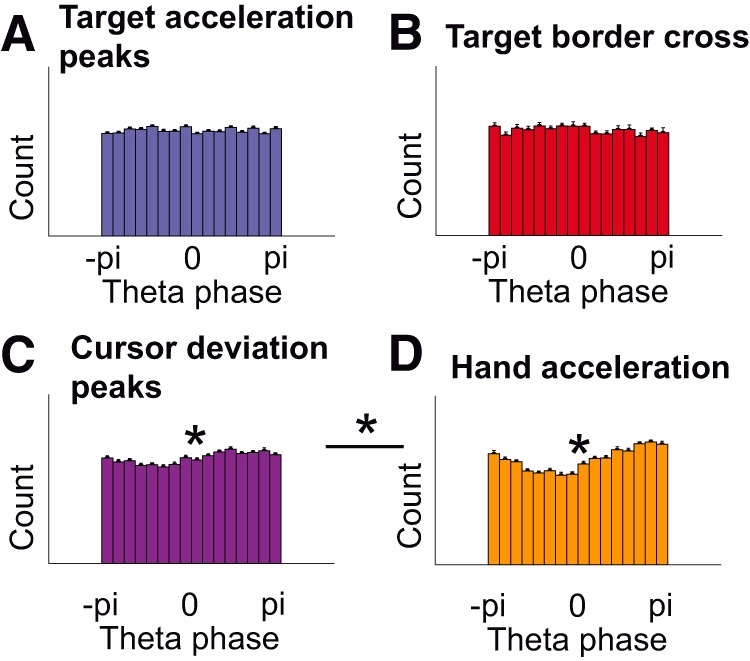
EEG phase-locking for different behavioral events. ***A***, No significant phase modulation was found for target acceleration peaks (zPLV = 0.09 ± 0.21, *p* = 0.67, *t*_(22)_ = 0.43, one-sample *t* test). Each histogram bar corresponds to the count of corresponding EEG phases at target acceleration peaks. ***B***, No significant phase modulation was found when the cursor left the target area (zPLV = 0.42 ± 0.36, *p* = 0.25, *t*_(22)_ = 1.17). ***C***, A weak but significant phase modulation was found for peaks in the Euclidian distance between cursor and target (zPLV = 1.50 ± 0.33, *p* = 0.00014, *t*_(22)_ = 4.60). ***D***, The largest (significant) phase modulation was found for submovements (peaks in hand acceleration; zPLV = 5.26 ± 0.64, *p* < 0.001, *t*_(22)_ = 8.18). This modulation was significantly stronger than for peaks in the Euclidian distance between the cursor and the target (***C***; *p* < 0.001, *t*_(22)_ = 6.86, paired *t* test).

Firstly, to verify that the coupling between the EEG and the kinematics was not due to indirect couplings with any visual event, we assessed phase-locking for four different types of events using zPLVs and phase histograms. No significant modulation was found for target acceleration peaks (zPLV = 0.09 ± 0.21, *p* = 0.67^j^, *t*_(22)_ = 0.43; Fig. 6*A*) nor when the cursor crossed the target borders from inside to outside the target (zPLV = 0.42 ± 0.36, *p* = 0.25^k^, *t*_(22)_ = 1.17; Fig. 6*B*). We found significant modulations for peaks in the Euclidian distance between the cursor and the target (zPLV = 1.50 ± 0.33, *p* < 0.001^l^, *t*_(22)_ = 4.60; Fig. 6*C*) and submovements (zPLV = 5.26 ± 0.64, *p* < 0.001^m^, *t*_(22)_ = 8.18; Fig. 6*D*). The phase-locking for submovements was significantly stronger than for peaks in the Euclidian distance (*p* < 0.001^n^, *t*_(22)_ = 6.86, paired *t* test). We thus confirm that, although peaks in the Euclidian distance between the cursor and the target modulate the phase of the EEG and could therefore lead to an ERP (Hill and Raab, 2005), the behavioral event leading to the strongest phase modulation were the submovements, in accordance with our analysis.

Secondly, since during tracking, the target speed corresponded to a smoothed copy of the hand kinematics from the spontaneous task, we controlled that our results corresponded to an action monitoring process of subjects’ own errors and not solely of unexpected target speed differences. We thus replicated the results from Fig. 5 in a control task during which 16 subjects tracked a target moving at constant speed along predefined trajectories (*N* = 20). Amplitudes were still significantly correlated to cursor deviation (*r* ^2^ = 0.976, *p* = 0.012^°^, *F*_(1,6)_ = 82.46; Extended Data Fig. 5-1).

10.1523/ENEURO.0241-17.2017.f5-1 Supplementary Figure 5-1 The peak-to-peak ERP amplitudes for the control experiment with constant target speed are in Figure 5-1A. Vertical black lines indicate target borders. Kinematics (top) and cursor deviation profiles corresponding to the ERP amplitudes are in Figure 5-1B. Download Figure 5-1, EPS file.

## Discussion

This study reports an ERP source-localized in the SMA and encoding behavioral deviations during continuous, visually-guided movements. The ERP was coupled to submovements defined by hand acceleration, a correlate of agonist/antagonist muscular activity (Vallbo and Wessberg, 1993). Phase-locking between the EEG and the submovements was much stronger compared to phase-locking with visual events such as target accelerations, the cursor leaving the target area or peaks in the Euclidian distance between target and cursor (Hill and Raab, 2005).

### Relation to cursor deviation

The amplitude of the ERP was positively correlated with the deviation of the cursor, 0.11 s before the submovement, thus 0.15 s before the ERP’s through. The more the cursor deviated behind the target, the larger the ERP and the acceleration of the submovements. When the cursor was in front of the target however, no discernible ERP was observed and submovement accelerations were similar. These results imply that the brain mechanism underlying the ERP does not encode an absolute value of the error such as the Euclidian distance but the amount of correction needed to catch up with the target. This modulation was also present, albeit much weaker when selecting similar submovements from the spontaneous task. This suggests that the cortical process underlying the ERP could be a hard-wired component of visually-guided movement loops, though not serving a functional purpose in artificial lab scenarios such as aimless (spontaneous) movements.

However, the modulation of the ERP by cursor deviation was still present in the tracking condition when selecting identical kinematics but different cursor deviations, excluding the possibility that the modulation due to cursor deviation is solely driven by differences in kinematics.

No significant ERP was found in the viewing condition, during which deviations were only observed with no motor output. The ERP is thus not generated by the visual content of the task stripped of its behavioral context. This does not eliminate the possible existence of an evaluative process in the brain such as in (van Schie et al., 2004), but not aligned to the same events (i.e. peaks in cursor acceleration).

### Relation to previous studies of action monitoring

The morphology and topography of the ERP shows clear similarities with ERP correlates of error found in discrete cognitive paradigms. The error-related negativity (ERN) is generated by the subjects’ own erroneous responses in speeded choice-response tasks, which do not require external sensory input to appraise the accuracy of the choice (Falkenstein et al., 1991; Gehring et al., 1993). The ERN peaks around 0.06 s after the motor response, a timing considered too early to rely on sensory feedback (Rodriguez-Fornells et al., 2002). The ERP in this study also peaks shortly after movement but its amplitude correlates with the cursor deviation. This suggests that it relies on visual feedback-related negativity (FRN) which is observed when errors are detected based on sensory feedback (Miltner et al., 1997).

Our analysis showed that this feedback is sampled 0.11 s before the submovement, inline with behavioral models suggesting that 0.115 s are enough to generate a corrective motor plan based on experimentally displaced cursor positions (Saunders and Knill, 2004).

Since the ERP occurs 0.038 s after the peak, this 0.11 s feedback sampling occurs around 0.15 s before the trough of the ERP, thus faster than the latency of the FRN (0.25–0.30 s after feedback onset). Interestingly, the continuous unfolding of the present task could allow for a better prediction of the feedback, thus allowing the brain to respond faster. Without motor output, error-related brain correlates do not scale to the magnitude of the error (Hajcak et al., 2006). However, similar scaling of the ERP amplitude by deviation/error can be seen when errors are motor-related (Vocat et al., 2011; Torrecillos et al., 2014).

These previous studies however, did not control for differences in hand kinematics. Moreover, there is a fundamental difference between behavior requiring one discrete response, or pointing hand movement, and continuous motor behavior, during which performance has to be constantly monitored. Our study can be seen as generalizing previous error-related ERP findings to the latter case, demonstrating that equivalent electrophysiological phenomena do actually operate in scenarios where feedback and behavior are not strictly experimentally segmented.

### Cortical network involved

For the tracking condition, the SMA was identified as the strongest source of the ERP. Previous studies have shown that the SMA is active during visually guided movements (Picard and Strick, 2003) and it’s activation is related to both submovement amplitude (Grafton and Tunik, 2011) and tracking error (Limanowski et al., 2017). Although the role of action monitoring was previously attributed to the anterior cingulate cortex (Ridderinkhof et al., 2004), a recent study showed that the activity of local field potentials in the SMA actually preceded activity in the anterior cingulate cortex when inhibiting a prepotent response (Bonini et al., 2014). This suggests that, consistent with our results, the SMA is involved in the recalculation of motor plans based on action monitoring. Interestingly, the precentral gyrus (were the motor cortex lies) was also part of the sources of the ERP and was found to be the strongest source of the ERP during the spontaneous condition. This suggest that the cortical network coupled to submovements is broader than the SMA, as was found in previous studies (Gross et al., 2002; Jerbi et al., 2007). Sources in the SMA were found only during tracking and not during spontaneous tracing, further supporting its involvement in action monitoring.

### Relation to low-frequency cortical oscillations

Our results show that an ERP is generated in synchrony with submovements. Considering the periodicity of the submovements, the ERPs should thus overlap with a periodicity of 5 Hz, therefore oscillating in the θ frequency band. Hence, it remains unknown whether the electrophysiological activity in this study corresponds to a succession of ERPs occurring at 5 Hz or oscillatory activity per se coupled to hand kinematics, as proposed in previous studies (Jerbi et al., 2007; Williams et al., 2010; Hall et al., 2014). θ (5 Hz) Oscillations are believed to support a number of cognitive operations (Cavanagh and Frank, 2014) such as memory encoding (Sederberg et al., 2003), error (Luu et al., 2004), response conflict (Cohen et al., 2008) or differences in decision confidence/threshold (Herz et al., 2016). θ Oscillations have also been linked to errors during tracking tasks (Huang et al., 2008; Cohen, 2016). Furthermore, modulations of attention have been found in the θ range in behavior (Fiebelkorn et al., 2013), EEG (Busch and VanRullen, 2010), and magnetoencephalography (Landau et al., 2015), leading to the hypothesis that θ represents the brain’s periodic attentional sampling mechanism (Fries, 2015; VanRullen, 2016). θ Periodicity found in active sensing behaviors (Schroeder et al., 2010) such as sniffing and whisking (Colgin, 2013) add support to this hypothesis. Our results can therefore also be interpreted within the framework of an oscillatory attentional process, periodically up-regulating cortical excitability to sample visual feedback at an optimal timing after the submovement. The network of low-frequency cortical oscillations coupled to hand kinematics (Jerbi et al., 2007) would thus serve to synchronize the periodic evaluation of cursor deviation to the motor output. It could be speculated that the periodicity of this brain mechanism could thus be scaled to match its capacity limit and explain the functional role of submovements in the framework of intermittent motor control (Craik, 1947; Neilson et al., 1988; Karniel, 2011). Interestingly, when varying the frequency of the display of visual feedback during a force tracking task, behavioral performance increased with increasing frequency of intermittent visual feedback up to 6.4 Hz and then reached an asymptote (Slifkin et al., 2000), supporting an optimal sampling of the visual feedback at θ frequency. Computational models have also approximated a capacity limit to be around 0.25 s (van de Kamp et al., 2013), corresponding to a θ rhythm. This hypothesis however, needs further experimental support.

In the future, the understanding of such mechanisms could improve the design of brain-machine interfaces (BMI), which decode brain signals to control a device, e.g. a prosthetic limb (Carmena et al., 2003; Hochberg et al., 2012). Electrophysiological signals such as the ERP in this study could be used to inform continuous BMI decoders about instant performance, as it has already been suggested for discrete paradigms (Chavarriaga et al., 2014).

## Synthesis

Reviewing Editor: Trevor Drew, University of Montreal Decisions are customarily a result of the Reviewing Editor and the peer reviewers coming together and discussing their recommendations until a consensus is reached. When revisions are invited, a fact-based synthesis statement explaining their decision and outlining what is needed to prepare a revision will be listed below. The following reviewer(s) agreed to reveal their identity: Nicholas Hatsopoulos, Roberto Caminiti

The authors have addressed all of the reviewers' concerns.
